# Improved pregnancy and birth rates with routine application of nonsurgical embryo transfer

**DOI:** 10.1007/s11248-014-9802-3

**Published:** 2014-05-06

**Authors:** Rahmen Bin Ali, Fina van der Ahé, Tanya M. Braumuller, Colin Pritchard, Paul Krimpenfort, Anton Berns, Ivo J. Huijbers

**Affiliations:** 1 MCCA Transgenic Core Facility, The Netherlands Cancer Institute, 1066 CX Amsterdam, The Netherlands; 2 Division of Molecular Genetics, The Netherlands Cancer Institute, 1066 CX Amsterdam, The Netherlands

**Keywords:** Nonsurgical, Embryo, Transfer, Implantation

## Abstract

Nonsurgical embryo transfer (NSET) of blastocysts to pseudopregnant female recipients provides many benefits over surgical implantation with less distress for the mice, no anesthesia or analgesia required and a considerable reduction in implantation time per mouse. Although a disposable device to perform NSET is on the market since 2009, it is not generally used in transgenic facilities, most likely because surgical implantation is efficient and inexpensive. Here, we report that with several refinements to the original protocol, the NSET method becomes very attractive and outperforms the traditional surgical transfer on basis of pregnancy rate, birth rate and implantation-related discomfort. Furthermore, repeated use of the same NSET device on several recipient females reduces the costs to a reasonable level. The data presented covers all embryo transfers over the last 5 years at the transgenic facility of the Netherlands Cancer Institute, of which the last 2 years were performed exclusively with NSET.

**Electronic supplementary material:**

The online version of this article (doi:10.1007/s11248-014-9802-3) contains supplementary material, which is available to authorized users.

Generating genetically engineered mice requires a diversity of skills and a considerable investment in time. Typically, embryonic stem cells (ESCs) are modified in vitro to introduce specific genetic alterations, after which the ESCs are injected into blastocysts. Next, the ESC-injected blastocysts are transferred in pseudopregnant female recipients in order to obtain chimeric mice with contribution of the modified ESCs to all tissues in order to achieve transmission of the genetic modifications to the next generation. All steps are crucial and need to be optimized. Recently, we have reported our efforts to optimize both the ESC culture conditions and the ESC microinjection method (Huijbers et al. 2014). Here, we focus on the transfer of ESC-injected embryos in female recipients. Currently, the golden standard to perform embryo transfer is surgical implantation. However, this is an invasive procedure and requires incisions through skin and muscle and penetration of the peritoneal cavity and reproductive tract. Furthermore, it requires externalization of internal organs and wound closure. The procedure is time consuming, requires significant technical expertise and necessitates anesthesia and analgesia in order minimize the discomfort of the foster mothers. In 2009, a less invasive nonsurgical embryo transfer (NSET) method was presented as an alternative and commercialized by ParaTechs (Lexington, KY, USA) (Green et al. 2009; Steele et al. 2013). This NSET method is based on the introduction of a disposable tapered Teflon catheter in vagina of the recipient female mouse, to expel manipulated embryos directly through the cervix into either one of the uterine horns of the mouse. The speed and simplicity of this process combined with the reduced burden/discomfort on the recipient mice made us adopt the NSET procedure for transfer of all our ESC-injected blastocysts from the beginning of 2012 onwards. In this report, we present our experiences and results during our transition from the surgical to the NSET procedure.

In 2011, we explored the NSET method as a possible replacement for the surgical embryo transfers by following the protocol provided with the NSET device. As shown in Table 1, we obtained similar pregnancy rates as compared to the surgical transfers performed in earlier years, however the percentage of live born mice was disappointing. By introducing further refinements, we achieved marked improvements, which were evident in the following years. The results even surpassed the efficiency of the surgical procedure on all fronts when comparing the combined figures of 2009–2010, i.e. surgical implantation only, versus the combined figures of 2012–2013, i.e. exclusively performed with the adapted NSET method (Table 1). Pregnancy rates increased from 76 to 85 %. Percentage of live born mice improved dramatically from 21 to 35 %. Furthermore, a complete absence of severe complications related to embryo transfer using the NSET method as opposed to 4 % with surgical implantation. The adapted NSET protocol is illustrated and explained in Fig. 1. Also a demonstration video is provided (Supplement video 1). The refinements that contributed to these encouraging results are explained in more detail below.

**Table 1:** Comparison between pregnancy rate, birth rate and implantation-related fatalities between surgical implantation and NSET

	Surgical		NSET		
	2009	2010	2011^a^	2012	2013
*Pregnancy rate*					
No. of implantations	52	66	143	190	146
No. of fosters used	159	196	432	584	328
No. of fosters pregnant	115	155	306	492	285
% Pregnancy	72.3	79.0	70.8	84.2	86.9
*Birth rate*					
No. of blastocysts transferred	1,787	2,232	6,698	8,783	5,615
No. of live-borns	350	485	669	3,125	1,845
% Live born pups	19.6	21.7	10.0	35.6	32.9
*Implantation-related fatality*					
No. of fosters died after transfer	6	7	0	0	0
% Fatality	3.8	3.6	0	0	0

^a^Start-up period in which several refinements were made to the original protocol

**Fig. 1:**
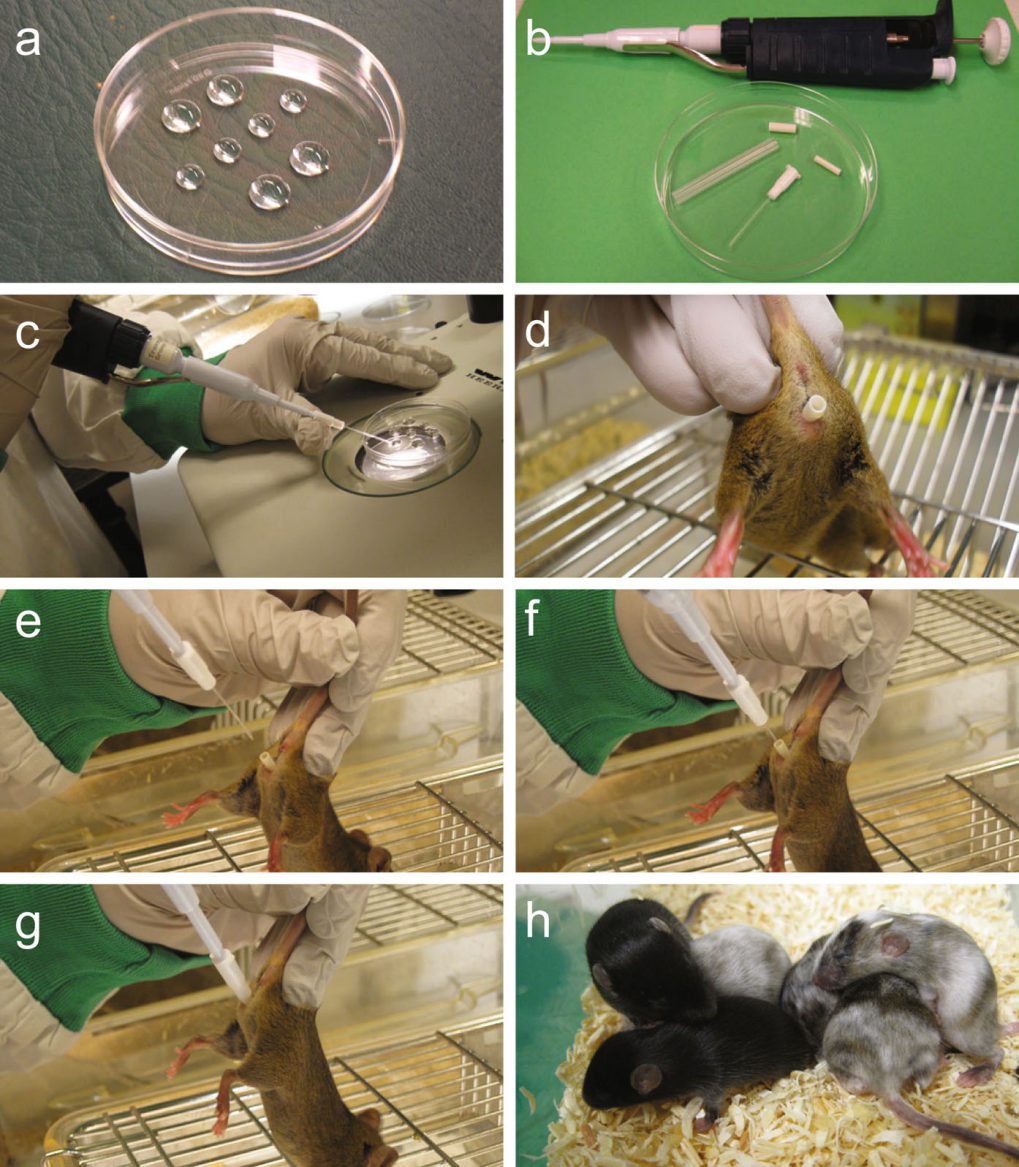
**a** Configuration of KSOM (Cat. No. MR-106-D; Millipore) drops on the cover of a 35 mm petridish (Falcon). The two drops in top row are used for consecutive washing steps of the ESC-injected embryos. Drops of second row contain the embryos to be transferred in a female recipient: each drop contains 15 blastocysts. The two drops in bottom row are used to rinse the NSET device after transfers. Volume of top and bottom drops is 100 μl and the middle drops 50 μl. Transfer of embryos between drops is performed by mouth pipette. **b** Materials required to perform NSET: a Gilson P2 pipette, the NSET device, a small and big speculum. Note, we omit the big speculum in our NSET protocol and exclusively use the small one. **c** Aspiration of embryos. Under a stereomicroscope the injected blastocysts are carefully aspirated with a catheter tip attached to a P2 pipette, dialed to 1.8 μl, until the first plunger stop. Next the pipette was dialed to 2.0 μl to create a small air bubble at the beginning of the tip. **d** Preparation of recipient mouse for effective transfer. Pick up the unanaesthetized pseudopregnant female mouse by the tail with forefinger and thumb and let her grab the metal grid with her forelimbs. Fix the body by placing the two free fingers at the bass of the tail. Carefully insert the small speculum into the vagina. **e** Positioning of the female for effective transfer. Lift the rear body of the mouse at an angle of 45°–70° in order to provide the most optimal entrance of the catheter tip in the cervix and uterine horn. **f** Introduction of NSET device. Pick up the NSET device loaded with 15 blastocysts and insert the tip in the speculum until it touches the edge of the speculum. **g** Embryo transfer. The blastocysts are released by pushing the plunger all the way till the second click. Without releasing the plunger, the NSET device is pulled out from the speculum. The speculum is removed and the mouse placed in a clean cage. **h** A typical litter obtained with NSET

(I) Avoid contamination with mineral oil. After microinjection, we pool the embryos in a drop of KSOM. This drop is covered with mineral oil to prevent evaporation. Before the transfer, the embryos are washed and transferred to separate drops of KSOM, with 15 blastocysts per drop (Fig. 1a). Occasionally, remnants of mineral oil are still present in these drops and can cause problems with the embryo transfer as mineral oil acts as a lubricant to the NSET catheter tip and can clog the catheter tip to prevent correct placement of embryos in the uterine horn. A complication also mentioned at the ParaTechs website. We simply avoid contact of the NSET catheter with the oil droplets when aspirating the injected blastocysts. Furthermore, we carefully rinse the catheter tip in clean drops of KSOM between transfers (Fig. 1a, bottom row) and check under the microscope whether the tip is sufficiently clean.

(II) Transfer 15 injected blastocysts per recipient female mouse. The protocol provided with the NSET device indicates a range of 12–20 embryos per recipient. Typically, we implant 15 embryos. We have transferred higher numbers, but then encountered more non-pregnant mice as well as fosters having difficulties giving natural birth. In cases where the mice did not deliver on due date, a caesarian section was performed a day later. Here, we often observed few, but large pups and embryo resorption in the uterus.

(III) Use the B6CBAF1/OlaHsd (Harlan) strain for the pseudopregnant recipients. Initially, we used the CD-1 strain (Charles River) as a recipient as suggested by ParaTechs, however we had difficulties to achieve successful entry into the uterine horn. We therefore, returned to using the strain we routinely used for our embryo transfer, i.e. the B6CBAF1/OlaHsd. The females on this background produce decent numbers of offspring and are caring mothers. Furthermore, the B6CBAF1/OlaHsd mice are generally leaner and calmer as opposed to CD-1. We start using the recipients when they reach 8 weeks of age and weigh between 20 and 35 g. Once the mice weight passes 35 g we no longer use them regardless of age.

(IV) Carefully select recipient female mice. One of the downsides of the NSET approach is the inability to evaluate whether a recipient female is stimulated or primed for implantation by observing the ovaries and uterus as can be done during surgical implantation. Transfer of embryos in unstimulated females will generally not yield successful pregnancies. We follow a set of simple rules for selecting appropriate recipient female mice suitable for the NSET method. First, only recipient female mice in their pro-estrous or estrous cycle are mated with vasectomised males as assessed by appearance of the vagina (Champlin et al. 1973). Second, we only implant in females with an evident vaginal plug. Third, we perform embryo transfer 2–3 h post blastocyst injection with ESCs. In brief, we select the appropriate recipient females and place them with vasectomised males at 3 p.m. The next day, we check for plugs at 8 a.m., i.e. 0.5 days post coitus (dpc). Two days later, ESC microinjection is performed in the morning and NSET in the afternoon between 2 and 3 p.m. In case of shortage of 2.5 dpc recipients on the day of embryo transfer, 3.5 dpc recipients can be used for the transfer of blastocysts. However, in this case NSET should be performed slightly earlier, between 12 a.m. and 1 p.m. In our hands, this works equally well.

(V) Position recipient females at the correct angle to allow undisturbed insertion of the NSET catheter tip. Select a recipient mouse in a calm state and let her grab the wire grid with the forelimbs and raise the back to insert the small speculum (Fig. 1d). Upon insertion of the NSET catheter tip, the body and speculum is positioned at an angle of 45°–70° (Fig. 1e). We find that this angle provides the best chance for the tip to glide through the cervix and into the uterine horn without hitting the cervix junction. In most cases, the insertion into the uterine horn will happen in one try, however if an obstruction is encountered, pull back the tip and try to reposition the tip and recipient before inserting it again. If the second try fails, try to use another recipient. Forcing your way through the cervix while trying to enter the uterine horn will cause the tip to bend and result in poor or no delivery of the blastocysts into the uterine horn and may cause injury to the mouse.

(VI) Only use the small speculum. The NSET protocol suggests to use first a small speculum to open the vagina followed by a larger speculum. We find that only using the small speculum to insert the catheter tip works equally well. Using sense of touch, the catheter tip is inserted through the small speculum and once it touches the edge of the speculum the tip has entered either one of the uterine horns (Fig. 1g). It is advisable to use a light source for guidance while inserting the device.

(VII) The same NSET device can be used for multiple transfers in 1 day. One of drawbacks for the NSET device is the cost involved. A box comes with ten devices, with a list price of $200–250 depending on the order size. ParaTechs strongly advices single use, which makes $20–25 per transfer. We routinely re-use our NSET devices for multiple transfers in 1 day. Some caution has to be taken. Avoid possible cross-contamination; only re-use the NSET device when the same type of ESC-injected blastocysts are to be implanted in multiple recipients. Between transfers, carefully clean the catheter tip by flushing with KSOM medium (Fig. 1a). It is good practice to check the condition of the catheter tip before and after each transfer under a stereomicroscope. Bent, dirty or blocked tips impair the efficiency of transfer and these should not be used. Under these conditions, we perform up to eight transfers with a single device, thereby substantially reducing costs.

(VIII) Practice makes perfect. Although the NSET method is straightforward and easy to learn, especially in comparison to the surgical implantation technique, still it requires experience to achieve the optimal results. In our first year of using the NSET method we observed reduced pregnancy rates and a strong drop in the number of live-born pups compared to the surgical transfer (Table 1). The refinements we subsequently introduced have undoubtedly contributed to the improved performance, however the increased experience in routinely performing the NSET method has also played a role. Currently, training of new personnel to perform the NSET method takes one or two sessions under supervision of a trained biotechnician. After that regular applying the technique is advisable for optimal results.

In conclusion, a range of relatively minor adaptations to the original NSET protocol improve the overall success rate of embryo transfer, even surpassing the generally used surgical embryo transfer. The adapted protocol is as simple as the original protocol and can easily be implemented in any transgenic facility. Our production data represent transfer of all our ESC-injected blastocysts over the last 5 years and do not include specific experiments to test the contribution of each individual adjustment to the protocol. We are therefore unable to state which adjustments provides most gain. In light of the 3R’s in animal research, i.e. replacement, reduction and refinement, the NSET method is a valuable “refinement” step as it causes less discomfort to the mice (Steele et al. 2013). It might also lead to a “reduction”, as improved embryo transfer reduces the number of fosters needed to obtain the required number of live born offspring, for instance when sanitizing or re-deriving strains. Recently, we ventured into Crispr/Cas9 injections in zygotes (Wang et al. 2013; Yang et al. 2013) and used both surgical and nonsurgical embryo transfer and found that the transfer of blastocysts derived from Crispr/Cas9 injected zygotes resulted in poor performance as compared to direct surgical implantation of the injected zygotes. Possibly, this is due to the delayed in vitro development of Crispr/Cas9 injected zygotes, as we have frequently observed. Therefore, implementation of the NSET method in transgenic facilities can provide considerable benefits, but should be applied correctly and in the appropriate setting.

## Electronic supplementary material

Below is the link to the electronic supplementary material.

Supplementary material 1 (MPG 43408 kb)
